# Performance and time to become negative after treatment of three malaria rapid diagnostic tests in low and high malaria transmission settings

**DOI:** 10.1186/s12936-016-1529-6

**Published:** 2016-10-04

**Authors:** Francesco Grandesso, Carolyn Nabasumba, Dan Nyehangane, Anne-Laure Page, Mathieu Bastard, Martin De Smet, Yap Boum, Jean-François Etard

**Affiliations:** 1 Epicentre, 8 rue Saint-Sabin, Paris, France; 2 Epicentre Mbarara Research Centre, Mbarara, Uganda; 3 Médecins Sans Frontières, Brussels, Belgium; 4 Mbarara University of Science and Technology, Mbarara, Uganda; 5 UMI 233 TransVIHMI, Institut de Recherche pour le Développement, Université de Montpellier 1, 34000 Montpellier, France

**Keywords:** Malaria, Fever, Diagnostic, Rapid diagnostic test, Sensitivity, Specificity, Performance, Negativity

## Abstract

**Background:**

The performance of different malaria rapid diagnostic tests (RDT) may be influenced by transmission intensity and by the length of time each test requires to become negative after treatment and patient’s recovery.

**Methods:**

Results of three RDTs (two HRP2 and one pLDH antigen-based tests) were compared to blood smear microscopy (the gold standard method) in children under 5 years of age living in a high versus low malaria intensity setting in southwestern Uganda. In each setting, 212 children, who tested positive by at least one RDT and by microscopy, were treated with artemether-lumefantrine. RDTs and microscopy were then repeated at fixed intervals to estimate each test’s time to negativity after treatment and patient recovery.

**Results:**

In the two settings, sensitivities ranged from 98.4 to 99.2 % for the HRP2 tests and 94.7 to 96.1 % for the pLDH test. Specificities were 98.9 and 98.8 % for the HRP2 tests and 99.7 % for the pLDH test in the low-transmission setting and 79.7, 80.7 and 93.9 %, respectively, in the high-transmission setting. Median time to become negative was 35–42 or more days for the HRP2 tests and 2 days for the pLDH test.

**Conclusions:**

High transmission contexts and a long time to become negative resulted in considerably reduced specificities for the HRP2 tests. Choice of RDT for low- versus high-transmission settings should balance risks and benefits of over-treatment *versus* missing malaria cases.

*Trial registration*: Registry number at ClinicalTrial.gov: NCT01325974

**Electronic supplementary material:**

The online version of this article (doi:10.1186/s12936-016-1529-6) contains supplementary material, which is available to authorized users.

## Background

Diagnosis of malaria based only on clinical grounds leads to significant overestimates of malaria illnesses even when diagnosis is carried out by experienced clinicians [1, 2]. Misdiagnosis is problematic for multiple reasons: it exposes patients to unnecessary side-effects of drugs and to the risk of overlooking potentially fatal conditions, such as pneumonia [3] or diseases of epidemic potential (i.e., Lassa fever or Ebola), and it contributes to the emergence of resistance to anti-malarials [4]. For these reasons the World Health Organization (WHO) recommends that every clinical suspicion of malaria should be confirmed by either microscopy or rapid diagnostic test (RDT) prior to initiating treatment [5]. Today RDTs have become the method of choice in many contexts since they allow an easy-to-use biological confirmation that can be performed in low-resource settings where good quality microscopy cannot be maintained, such as remote health centres or at community level.

The number of different RDTs on the market has increased considerably over the past few years. In 2008, WHO launched the Product Testing Programme, which evaluates performance of commercialized malaria RDTs against a standardized panel of laboratory-prepared specimens. This programme, which has already completed its fifth round of testing, allows for product comparisons, encourages producers to improve their tests and, in the long run, promotes product quality. WHO acknowledges however, that its results cannot predict test performance in the field, where other factors such as malaria antigen persistence in the blood and malaria endemicity can influence RDT performance [6].

Most malaria RDTs are designed to detect a single parasite antigen, the histidine-rich protein 2 (HRP2) or the *Plasmodium* lactate dehydrogenase (pLDH), while others are designed to detect both antigens in a single test.

HRP2 is a protein produced only by the *Plasmodium falciparum* species; detection of this protein can therefore confirm only *P. falciparum* infections. Many of the HRP2-based RDTs show very high scores in the WHO evaluation [7]. For instance, the CareStart HRP2 was reported to have a 98.7 % detection rate (a proxy measurement of sensitivity) and a false positive rate (proxy of the inverse of specificity) of 2.4 %. However, these HRP2-based tests performed more poorly in the field, particularly in terms of their specificity, due primarily to the slow elimination of the HRP2 antigen from the bloodstream. This persistence of the HRP2 antigen means that patients continue to test positive with HRP2-based RDTs long after the patient was treated and the parasites eliminated. During this period, these tests therefore cannot distinguish between lingering positivity and de novo infection. This phenomenon is well documented in high-transmission areas where patients frequently experience repeated malaria infections and treatments [8–10]. However, little is known about field performance of these tests in low-transmission areas where malaria infections are rare.

The pLDH is a parasite protein produced by all *Plasmodium* species; RDTs based on this antigen can therefore detect all *Plasmodium* species but are generally less sensitive than HRP2 tests. Their main advantage is that pLDH is rapidly eliminated from the blood stream after treatment, and therefore pLDH-based RDTs returns a negative result within a few days after treatment.

The objective of this study was to explore whether and how much three malaria RDTs vary, in terms of sensitivity, specificity and time to become negative, based on transmission setting. The three RDTs chosen were among the highest scoring tests in the WHO Round 3 malaria test performance evaluation [7] (two HPR2 RDTs and one pLDH RDT). Tests were assessed in parallel in two settings: one with high malaria transmission, the other with low transmission, in southwestern Uganda.

## Methods

### Study settings

The study was conducted from September 2011 to January 2013 within the Greater Mbarara district at two locations: Mbarara municipality, where malaria prevalence in children below 5 years of age was relatively low (3–4 %), and Kazo, located 80 km north of Mbarara municipality, where malaria prevalence was ten-fold higher (40–60 %) as reported in a previous study [11]. In Mbarara municipality the study was hosted at the Kakiika Health Centre; seven other health structures located within or just outside the Mbarara municipality also participated by providing eligible patients. In Kazo the study was hosted at the sole health centre present in the sub-county.

The Greater Mbarara district is located between 130 m and 1500 m altitude above sea level and its vegetation is a mixture of bush and short grass. The climate is tropical with a bi-modal rainfall pattern averaging 1200 mm per annum. The rainfall season falls between mid-August to December and mid-February to May. Malaria transmission is on-going throughout the year, with two seasonal peaks occurring 2 months after the periods of heavy rainfall. *Plasmodium falciparum* is almost the only species documented. Malaria constitutes one of the major causes of illness in the area.

### Sample size

The sample size was calculated to respond to the main study objective, the time to become negative of RDTs, and was based on the expected proportions of RDTs that remained positive at each follow-up day. It was estimated that 184 patients were needed to achieve the accuracy of 8 % around a proportion of 50 % positive RDTs and of 3 % around proportions of 5 or 95 %. By expecting 15 % of patients not being positive for all RDTs, lost to follow up or excluded from the primary analysis, 212 patients were estimated to be included in each study site.

For the sensitivity analysis all patients with a positive microscopy with a *P. falciparum* infection were included until the sample size for the time to become negative analysis was achieved. For the specificity analysis, the number of patients with a negative microscopy depended of the intensity of malaria transmission. A negative/positive microscopy ratio of around 1:1 and of at least 5:1 was expected in the high and low transmission settings respectively.

### Patient recruitment and follow-up

The study recruited consecutively all children who presented to any of the participating health centres, were under 5 years of age, weighed 5 kg or more, and either had fever ≥37.5 °C or whose parent reported fever within the previous 2 days. Blood samples obtained by finger prick from children whose parent or guardian provided written consent were tested in parallel at the corresponding study health centre with the three different RDTs and with microscopy. Patients recruited at this stage formed the population sample for the RDT performance analysis.

At each site, a sub-set of 212 children was selected for the time to become negative analysis. Inclusion criteria were: positivity for at least one RDT, having a *P. falciparum* mono-infection (i.e., no additional species) confirmed by microscopy, and having not taken any anti-malarial drug within the previous 2 weeks (as reported by the parent). Participants stayed with a parent or guardian in an observation room of the health centre for 3 days (day 0–day 2) to receive a full course of artemether-lumefantrine (Coartem®, Novartis) under the supervision of study personnel. When treatment intake was completed, participants were sent home. During their stay at the health centre and at follow-up visits on a fixed schedule (days 2, 3, 5, 7, 14, 21, 28, 35, and 42) patients received a clinical check-up and malaria testing using all three RDTs and microscopy. If an RDT was negative at a given visit, that test was not repeated during subsequent visits. Follow-up was discontinued as soon as all RDTs were negative, or at a maximum of 42 days.

Patients with recurrent parasitaemia during follow-up were treated with artesunate-amodiaquine (AS–AQ Winthrop®, Sanofi Aventis). Patients who did not appear for a follow-up visit after day 3 (when they went home from the centre) and for whom it was not possible to recover any additional information were considered lost to follow-up.

### Laboratory techniques

The following RDTs were evaluated: SD Bioline Malaria Antigen P.f (HRP2) (Standard Diagnostic Inc, Suwon, South Korea, catalogue number: 05FK50-02-4), CareStart Malaria HRP2 (Pf) (Access Bio, Somerset, USA, catalogue number: G0141) and CareStart Malaria pLDH (PAN) (Access Bio, catalogue number: G0111). RDTs were performed according to manufacturer’s instructions. All RDTs were read by two blinded, independent readers. The first reading was done at 15 min for the SD Bioline test and 20 min for the two CareStart tests. The first reader wrote the result in a paper form which was handed to the lab supervisor. The supervisor called within 5 min the second reader who wrote the result in a second paper form. The supervisor confronted the results. If results were discordant the supervisor acted as third tie-breaker reader. The third reading occurred typically within 10 min after the first reading. The lot number of each test was recorded and the storage temperature monitored.

For microscopy, thick and thin blood smears were prepared on the same slide and stained with a 10 % Giemsa solution (pH 7.2) for 15 min. Reading was performed using a 100× magnification lens with oil immersion. Parasite asexual forms were counted against a threshold of 200 white blood cells (WBC); if fewer than ten parasites were seen at this threshold, counting was continued until at least 500 WBC. *Plasmodium* species was confirmed on the thin smear. Two-hundred high-power fields were read before declaring a blood slide negative.

Parasite density was estimated based on a hypothetical leukocyte density of 8000 WBC/µL. The presence of gametocytes was recorded, although a slide with gametocytes but no asexual parasite forms was scored as negative.

Double reading of all slides was performed. Slides with discordant results (positive/negative or parasite density of second reader varying by ≥50 % compared to the first reader) were read by a third reader. The parasite density result was calculated as the mean of the first and second readers, if not discordant, or as the mean of the two closest results, if a third reading was required. Blinding of microscopists to RDT results was ensured by assigning to the slides a different laboratory code, so that only the laboratory supervisor could match the microscopy result to the patient’s identification number that was used for the RDT. All study microscopists had a Kappa score ≥80 % in evaluations of microscopists conducted by the reference laboratory of the Shoklo Malaria Research Unit (Thailand) in 2010, prior to the start of this study.

### Data processing and analysis

Data were double-entered and validated in a Voozanoo database (EpiConcept, Paris, France). Data analysis was carried out using Stata version 12.1 (Stata Corp, College Station, TX, USA).

Qualitative variables were assessed by percentages and 95 % confidence intervals (95 % CI). Quantitative variables were described by mean and standard deviation if normally distributed, and by median and interquartile 25 and 75 percentiles range (IQR), if not normally distributed. Comparison of continuous numeric variables was tested with Student *t* test if normally distributed, and with Mann-Whitney rank-sum test if not normally distributed.

Sensitivity, specificity, positive and negative predictive values, and misclassification rates were estimated for each RDT separately, with microscopy as the gold standard. All patients with *P. falciparum* (mono- or mixed species infection) were included in the analysis, while patients with an infection that did not include *P. falciparum* were excluded, since the HRP2 tests in the study detect only *P. falciparum*.

RDT sensitivity and specificity were defined as the proportion of positive test results among the microscopy-positive blood smears, and the proportion of negative tests among the negative blood smears, respectively. The positive and negative predictive values were defined as the proportion of positive blood smears among the positive tests, and of negative blood smears among the negative tests, respectively. The misclassification rate was defined as the proportion of erroneous results (the sum of false positive and false negative results) among the total number of tests performed.

Performance comparisons between RDTs in the same site, and of the same RDT among sites, were expressed as differences with 95 % CI. McNemar’s test was used to assess statistical significance in performance among RDTs in the same site while Fisher’s exact test was used to assess statistical significance in performance of the same RDT between sites.

Sensitivity of RDTs in three strata defined as low (1–1999 parasites/µL), medium (2000–199,999 parasites/µL) and high parasite density (200,000 parasites/µL or more) were also assessed and compared. For this last analysis, results from the two sites were combined.

The time to become negative was defined as the 1st day when an RDT was reported as negative. Patients with incomplete or incorrect intake of anti-malarial treatment and patients who presented recurrent parasitaemia starting from day 7 (i.e., malaria microscopy was again positive) before all RDTs had become negative were excluded from the analysis. Patients lost to follow-up were censored at their last visit.

The probability of a test remaining positive over time was calculated with the Kaplan–Meier survival function. The same survival analysis was carried out on three strata according to initial parasitaemia. Strata were defined as low, medium and high parasite densities (same cut-off as for RDT performance).

### Ethics, consent and permissions

The study protocol was approved by the Ethical Review Board of *Médecins Sans Frontières*, the Faculty Research Ethical Committee and the Institutional Review Board of Mbarara University of Sciences and Technology, and the Uganda National Council for Sciences and Technology.

All patients were recruited after a parent or guardian provided written consent, and all patients received free health care during the entire period of study participation.

## Results

### Patient characteristics

Recruitment in the Mbarara municipality took place from November 2011 until the end of January 2013, an extended period that reflected the area’s low malaria transmission, and consequently, the long time needed to recruit sufficient numbers of malaria-positive patients. Patients in high-transmission Kazo were recruited from September to December 2011.

**Fig. 1:**
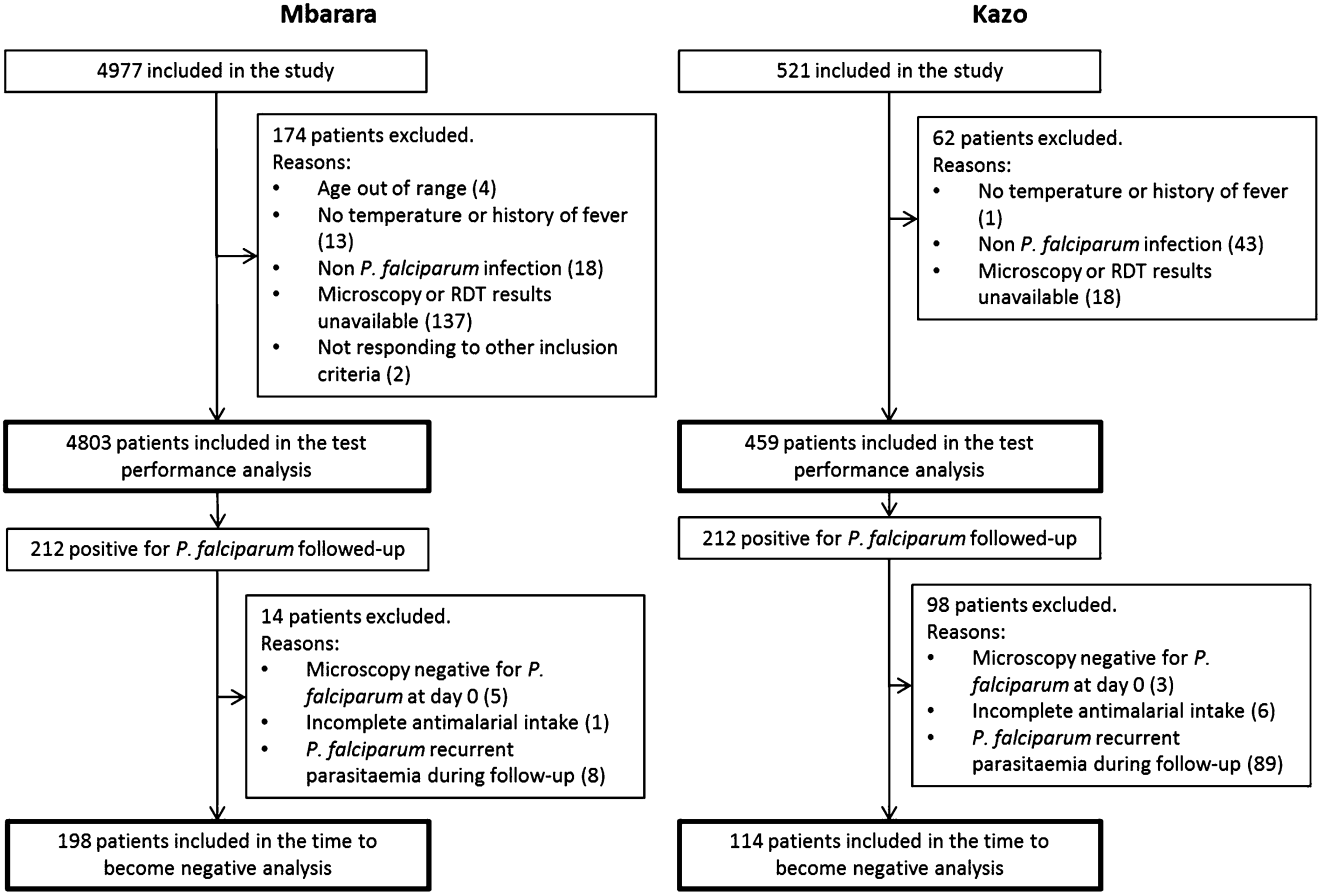
Flowchart of patients participating to the study

A total of 4977 and 521 patients were admitted to the study in Mbarara and Kazo, respectively, of whom 4803 and 459 patients were included in the RDT performance analysis. The reasons for exclusion are listed in (Fig. 1). The number of invalid tests was 121 for SD Bioline HRP2, three for CareStart HRP2 and one for CareStart pLDH. The median age of participating children in Mbarara was significantly lower (17 months) than in Kazo (24 months) (p value <0.001) while haemoglobin level was higher in Mbarara than in Kazo (Table 1).

**Table 1:** Demographic and clinical characteristics of children participating in the study

Included in the performance analysis (N)	Mbarara		Kazo	
	4803		459	
Gender				
Male (n, *%*)	2493	51.9	218	47.5
Female (n, *%*)	2310	48.1	241	52.5
Age (months) (median, IQR)^$^	17	10–31	24	13–36
Reported fever (n, *%*)	4783	99.8	446	97.4
Temperature >37.5 °C (n, *%*)	2296	47.9	233	51.0
Haemoglobin (g/dL) (mean, SD)^§^	11.4	1.6	10.3	2.1
Blood smear positive for *P. falciparum* (n, *%*)	256	5.3	262	57.1

Included in the time to become negative analysis (N)	Mbarara		Kazo	
	198		114	
Gender				
Male (n, *%*)	95	48.0	51	44.7
Female (n, *%*)	103	52.0	63	55.3
Age (months) (median, range)^‡^	29.5	15–44	23	12–33
Reported fever (n, *%*)	197	99.5	111	97.4
Temperature >37.5 °C (n, *%*)	142	71.7	74	69.2
Haemoglobin (g/dL) (mean, SD)^£^	10.4	1.9	10.1	2.0
*P. falciparum* asexual forms parasites per µL (median, IQR)^¥^	48,528	8473–132,000	37,962	6545–125,333

^$^ Mann–Whitney rank-sum test p value <0.001

^§^ Student’s* t*-test p value <0.001

^‡^ Mann–Whitney rank-sum test p value = 0.003

^£^ Student’s* t*-test p value = 0.184

^¥^ Mann–Whitney rank-sum test p value = 0.573

Of the 212 patients recruited at each site for evaluating each RDT in terms of time to negativity after malarial treatment, 14 (6.6 %) and 98 (46.2 %) patients in Mbarara and Kazo, respectively, were excluded from the analysis at some point before completion of their planned visits. The most common reason for exclusion was the occurrence of a recurrent parasitaemia during follow-up, which was found in eight patients (3.8 %) in Mbarara and 89 patients (42 %) in Kazo. Other reasons are listed in the study flow chart (Fig. 1). The analysis was ultimately carried out on a sample of 198 and 114 patients in Mbarara and Kazo respectively. In this participant group, the median age was higher in Mbarara (29.5 months) than in Kazo (23 months) (rank-sum test p value = 0.003). Median levels of parasitaemia were 48,528 and 37,962 parasites/µL in Mbarara and Kazo, respectively, with no significant difference between the two study sites (rank-sum test p value = 0.573) (Table 1). The number of *P. falciparum* mono-infections, mixed and non-falciparum infections are listed in Table 2.

**Table 2:** *Plasmodium* species in patients included in the RDT performance analysis by study site

	Mbarara		Kazo	
	n	%	n	%
Total patients positive (any *Plasmodium* species)	285	100.0	306	100.0
*P. falciparum* mono-infection	260	91.2	233	76.1
Mixed *P. falciparum* infection	7	2.5	30	9.8
Other non-*P. falciparum* mono-infections	18	6.3	43	14.1
*P. vivax* mono-infection	2	0.7	1	0.3
*P. ovale* mono-infection	4	1.4	10	3.3
*P. malariae* mono-infection	12	4.2	32	10.5

### Test performance

RDT performance data in the different settings are summarized in Table 3. The sensitivities of the two HRP2 tests ranged from 98.4 to 99.2 %, with no significant difference between the two RDTs and between sites. Sensitivity of the pLDH test, which was 96.1 % in low-transmission Mbarara and 94.7 % in high-transmission Kazo, was significantly inferior to that of CareStart HPR2 in Mbarara (McNemar p value: 0.0156) and to both SD Bioline HRP2 and CareStart HRP2 in Kazo (McNemar p values: 0.0005 and 0.0034, respectively). In five patients, three in Mbarara and two in Kazo, the microscopy was positive for *P. falciparum* but all three RDTs were negative. The parasite densities were low or moderated for four of these patients (110, 246, 1081 and 10,805 parasites/µL) and very high for one patient (282,667 parasites/µL). Specificities of all three RDTs were high in Mbarara, ranging from 98.8 to 99.8 %. In Kazo, the specificities were 79.7 % for SD Bioline HRP2 80.7 % for CareStart HPRP2 and 93.9 % for CareStart pLDH, which were significantly inferior to Mbarara, especially for the two HRP2 tests (19.2 % for SD Bioline HRP2 and 18.1 % for CareStart HPRP2). A less marked, though still significant, inferior specificity (5.8 %) was observed for the CareStart pLDH.

**Table 3:** Performances and differences in performances among study sites and RDTs

Test parameter	Site	SD Bioline HRP2		CareStart HRP2		CareStart pLDH		SD Bioline HRP2 *vs* CareStart HRP2		SD Bioline HRP2 *vs* CareStart pLDH		CareStart HRP2 *vs* CareStart pLDH	
		%	[95 % CI]	%	[95 % CI]	%	[95 % CI]	Diff %	[95 % CI]	Diff %	[95 % CI]	Diff %	[95 % CI]
Sensitivity	Mbarara	98.4	[96.0 to 99.6]	98.8	[96.6 to 99.8]	96.1	[92.9 to 98.1]	−0.4	[−1.5 to 0.8]	2.3	[−0.2 to 4.9]	2.7	[0.3 to 5.1]*
	Kazo	99.2	[97.3 to 99.9]	98.9	[96.7 to 99.8]	94.7	[91.2 to 97.0]	0.4	[−0.7 to 1.5]	4.6	[1.7 to 7.5]***	4.2	[1.2 to 7.2]**
	Diff	−0.8	[−2.6 to 1.0]	0.0	[−1.9 to 1.8]	1.4	[−2.2 to 5.0]						
Specificity	Mbarara	98.9	[98.5 to 99.2]	98.8	[98.5 to 99.1]	99.8	[99.6 to 99.9]	0.0	[−0.1 to 0.2]	−0.9	[−1.2 to −0.6]***	−0.9	[−1.2 to −0.6]***
	Kazo	79.7	[73.4 to 85.1]	80.7	[74.5 to 86.0]	93.9	[89.6 to 96.8]	−1.0	[−2.9 to 0.9]	−14.2	[**−**19.8 to −8.6]***	−13.2	[−18.6 to −7.8]***
	Diff	19.2	[13.6 to 24.8]^$$$^	18.1	[12.6 to 23.6]^$$$^	5.8	[2.5 to 9.2]^$$$^						
Misclassification	Mbarara	1.1	[0.9 to 1.5]	1.2	[0.9 to 1.5]	0.4	[0.3 to 0.7]	0.0	[−0.1 to 0.1]	0.7	[0.4 to 1.0]***	0.7	[0.4 to 1.0]***
	Kazo	9.2	[6.7 to 12.2]	8.9	[6.5 to 11.9]	5.7	[3.7 to 8.2]	0.2	[−0.7 to 1.2]	3.5	[0.5 to 6.5]*	3.3	[0.3 to 6.2]*
	Diff	−8.0	[−10.7 to −5.4]^$$$^	−7.8	[−10.4 to −5.1]^$$$^	−5.2	[−7.4 to −3.1]^$$$^						
Positive predictive value	Mbarara	83.2	[78.5 to 87.2]	82.7	[70.0 to 86.7]	95.7	[92.5 to 97.8]	–		–		–	
	Kazo	86.7	[82.3 to 90.3]	87.2	[82.9 to 90.8]	95.4	[92.1 to 97.6]	–		–		–	
	Diff	−3.5	[−9.2 to 2.2]	−4.5	[−10.2 to 1.2]	0.3	[−3.2 to 3.9]						
Negative predictive value	Mbarara	99.9	[99.8 to 100.0]	99.9	[99.8 to 100.0]	99.8	[99.6 to 99.9]	–		–		–	
	Kazo	98.7	[95.5 to 99.8]	98.1	[94.7 to 99.6]	93.0	[88.5 to 96.1]	–		–		–	
	Diff	1.2	[−0.6 to 2.9]^$^	1.8	[−0.3 to 3.9]^$^	6.8	[3.3 to 10.4]^$$$^						

* Exact McNemar’s p value <0.05

** Exact McNemar’s p value <0.01

*** Exact McNemar’s p value <0.001

^$^ Fisher’s exact p value <0.05

^$$^ Fisher’s exact p value <0.01

^$$$^ Fisher’s exact p value <0.001

The positive predictive values of the two HRP2 tests were similar in Mbarara and Kazo. The negative predictive value of the pLDH test was lower in Kazo (but not in Mbarara) compared with the HRP2 tests.

Variation in RDT performance based on parasite density stratification was similar at the two study sites (Additional file 1) and were therefore pooled from both study sites. The sensitivity in patients with low parasite density was 94.5 % for both HRP2 tests and 75.3 % for CareStart pLDH (McNemar’s p values <0.001 for CareStart pLDH versus both HRP2 tests), while the sensitivity of all three RDTs ranged between 98.3 and 99.7 % in the medium and high parasite density strata, with no difference between these two latter strata and among RDTs (Table 4). One patient with a parasite density above 200,000 parasites/µL had a negative result with all three RDTs evaluated.

**Table 4:** Sensitivity of three RDTs stratified by parasite density (Mbarara and Kazo study sites combined)

Parasite density groups	SD Bioline HRP2		CareStart HRP2		CareStart pLDH		SD Bioline HRP2 *vs* Carestart HRP2		SD Bioline HRP2 *vs* CareStart pLDH		CareStart HRP2 *vs* CareStart pLDH	
	%	[95 % CI]	%	[95 % CI]	%	[95 % CI]	Diff %	[95 % CI]	Diff %	[95 % CI]	Diff %	[95 % CI]
Low (1–1999 parasites/µL) N = 73	94.5	[86.6 to 98.5]	94.5	[86.6 to 98.5]	75.3	[63.9 to 84.7]	0.0	[−5.2 to 5.2]	19.2	[8.0 to 30.3]***	19.2	[8.0 to 30.3]***
Medium (2000–199,999 parasites/µL) N = 386	99.7	[98.6 to 100]	99.7	[98.6 to 100]	98.7	[97.0 to 99.6]	0.0	[−0.3 to 0.3]	1.0	[−0.2 to 2.3]	1.0	[−0.2 to 2.3]
High (200,000 + parasites/µL) N = 59	98.3	[90.9 to 100]	98.3	[90.9 to 100]	98.3	[90.9 to 100]	0.0	[−1.7 to 1.7]	0.0	[−1.7 to 1.7]	0.0	[−1.7 to 1.7]
Difference between parasite density groups												
Low–medium	−5.2	[−10.5 to 0.0]^$$^	−5.2	[−10.5 to 0.0]^$$^	−23.4	[−33.3 to −13.4]^$$$^						
Low–high	−3.8	[−10.0 to 2.4]	−3.8	[−10.0 to 2.4]	−23.0	[−33.4 to −12.5]^$$$^						
Medium–high	1.4	[−1.9 to 4.8]	1.4	[−1.9 to 4.8]	0.4	[−3.1 to 3.9]						

* Exact McNemar’s p value <0.05

** Exact McNemar’s p value <0.01

*** Exact McNemar’s p value <0.001

^$^ Fisher’s exact p value <0.05

^$$^ Fisher’s exact p value <0.01

^$$$^ Fisher’s exact p value <0.001

### Time to become negative

**Fig. 2:**
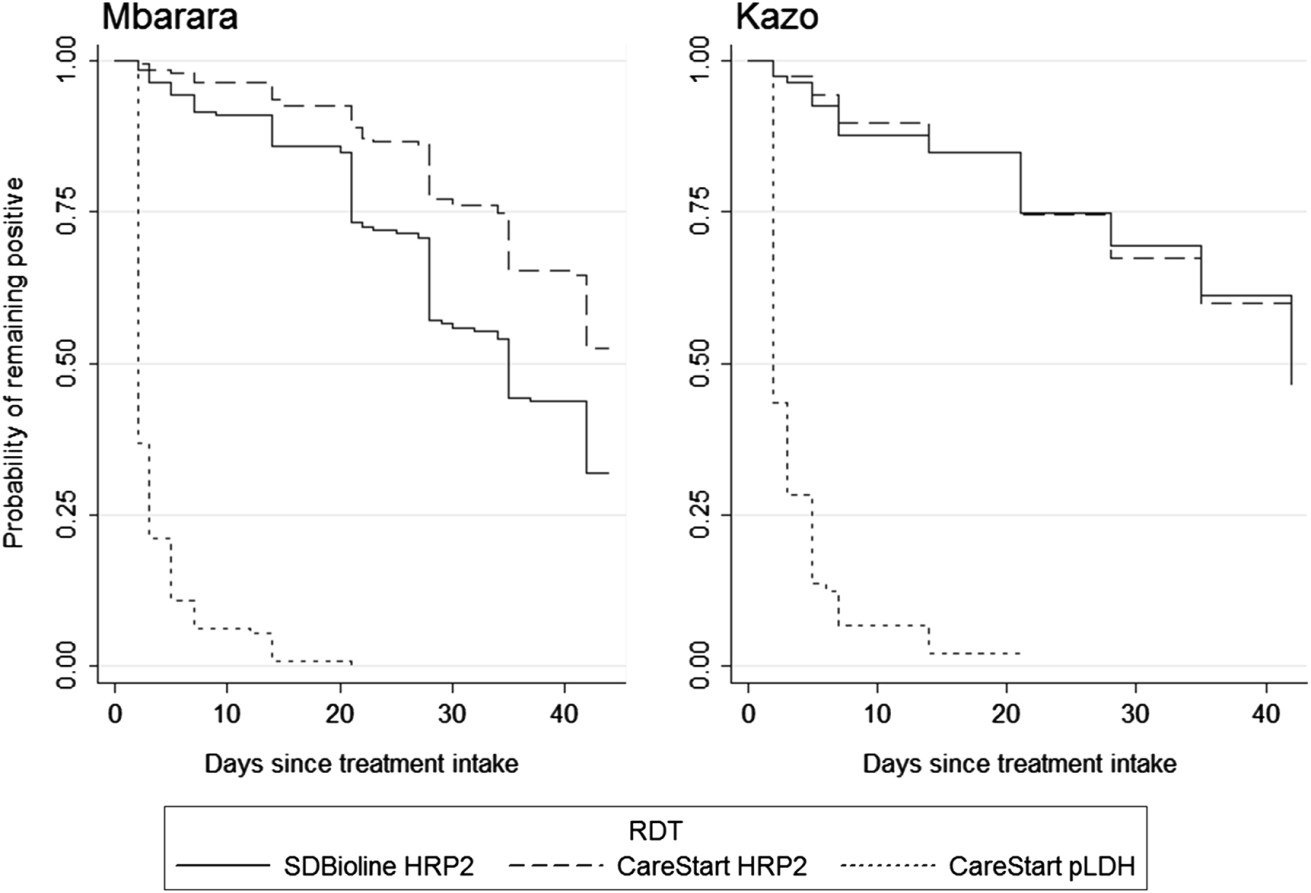
Time to become negative for three malaria RDTs, expressed as Kaplan–Meier survival estimates, in Mbarara (low transmission setting) and in Kazo (high transmission setting)

**Fig. 3:**
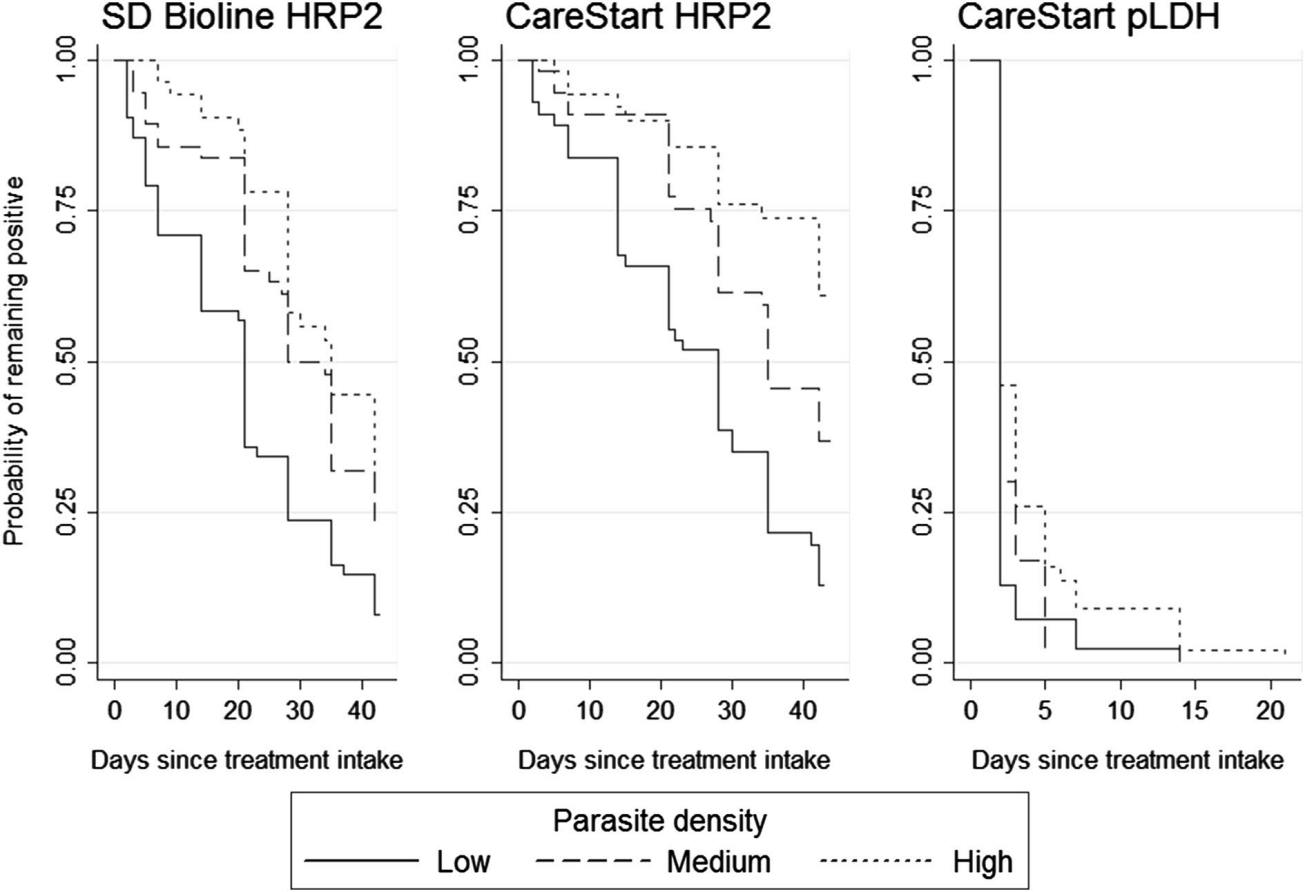
Time to become negative for three RDTs stratified by parasite density at patient’s admission, expressed as Kaplan–Meier survival estimates. Patients from Mbarara (low-transmission setting) and Kazo (low transmission setting) are combined

The median time to become negative was 35 and 42 days for the SD Bioline HRP2 in Mbarara and Kazo, respectively, and 2 days for the CareStart pLDH at both sites. For the CareStart HRP2 test, the median time could not be calculated because it exceeded 42 days, the maximum follow-up time for patients in this study (Fig. 2). For the same reason, it was possible to calculate the 95th percentile only for the pLDH tests, which was 14 days. In both settings patients with higher parasitaemia at inclusion had a longer time to become negative (Fig. 3). Similar results were found in the analysis when patients with recurrent parasitaemia were censored at the day when the recurrent parasitaemia occurred (Additional file 2).

## Discussion

All three RDTs evaluated in this study performed better in the low- than in the high-transmission setting, with striking differences especially regarding specificity. While the low specificity of the HRP2 tests (around 80 %) in the high-transmission setting of Kazo confirmed previous studies from similar epidemiological contexts, for example, 73 % in Ghana [12] and 63 % in Tanzania [13], the high specificity (almost 99 %) in the low-transmission setting of Mbarara municipality was consistent with the very low false positive rate reported in the WHO Product Testing Programme [7].

The long-lasting HRP2 antigens, when combined with a high frequency of malaria infections, may lead to quasi-persistent antigen positivity in children living in high malaria transmission areas. A clear demonstration of this situation is the high number of children (86 out of 212) in Kazo that experienced a recurrent malaria infection before clearing the HRP2 antigens of the previous one. This is not the case for children living in low-transmission settings, who are much less likely to have had a recent malaria infection.

Unlike specificity, the sensitivity of all three RDTs was high in both settings; however the pLDH test performed less well than the HRP2 tests. Sensitivity estimates confirm the WHO evaluations [6, 7] and are in line with previous studies [14, 15]. Only Nyunt and colleagues found that the sensitivity of the pLDH test was as high as an HRP2 test [10].

Data of this study clearly indicate that the relatively low sensitivity of the pLDH test was observed only at low parasite density. Nevertheless, this low sensitivity is highly problematic for public health decision makers, who typically favour deploying highly sensitive tests such as the HRP2 RDTs, accepting the associated risk of unnecessarily treating uninfected patients over taking the risk of failing to detect malaria cases. Regarding specificity, the high number of false positives observed with HRP2 tests is also problematic, particularly in high transmission settings where half or more of RDTs are positive. A wrongly positive result may divert clinicians’ attention from other possible aetiologies of febrile illnesses, or may lead clinicians to mistrust the RDT result, diminishing their perceptions of the test’s usefulness. The two HPR2 tests showed much longer times to become negative (median of 35 to >42 days) than expected from most previous reports. Only one study in the Democratic Republic of Congo [9] reported such a long time to become negative, whereas others report median times of no longer than 21 days [6, 14, 16].

In contrast, the two-day median time of pLDH antigen persistence generally seems to parallel a decline in the number of viable parasites during treatment [15, 17, 18]. In some instances, however, the pLDH antigen may persist for longer. Nyunt and colleagues reported in their study in Myanmar a median time of 6 days [10]. Njama-Meya and colleagues reported that 7 % of patients were still positive at 28 days. In this study no patient was positive beyond 21 days. The reasons for this difference remain unexplained.

While time to become negative did not vary substantially according to the intensity of transmission, it differed based on initial parasite density: the higher the initial parasite density, the more time was required to clear parasite antigens [8]. This finding supports the hypothesis that the level of antigens in the bloodstream is proportional to parasite density [19].

The study had a number of limitations. Some negative microscopy results may represent real infections with a sub-microscopic parasite density. In this case, if the RDT was positive, results may have been wrongly classified as false positive instead of true positive. Considering the high sensitivity of HRP2 tests in low parasitaemias, this circumstance may have affected a higher proportion of HRP2 test results than pLDH results.

Double reading of all slides and the inspection of 200 fields before scoring a blood smear as negative were performed to ensure high quality results. Nevertheless, it may be possible that some low parasite densities went undetected and were therefore wrongly classified as negative [20].

The PCR molecular assay is proved to be more sensitive than a well performed microscopy [21] and would have provided more comparable results with recently published studies [22]. Nevertheless, while the high sensitivity provided by the PCR is important in contexts where it is crucial to detect infections with low parasite density—e.g. in a malaria elimination programme—its added value in clinical diagnosis is arguable, since the clinical meaning of a child’s sub-microscopic infection in a high-transmission area is still debated.

Children with recurrent parasitaemia during follow-up were excluded from the time to become negative analysis. This choice had the consequence of reducing the size of the analysable patient cohort. Reduction was limited in the low-transmission setting (eight patients in Mbarara), but led to exclusion of one-third of all patients at the Kazo site (89 patients). The characterization of polymorphic markers to distinguish a re-infection from a recrudescence, a standard method in anti-malarial efficacy studies, would have allowed to censor, rather than to exclude, these children.

The time to become negative for a given RDT was determined on the basis of the first negative result during follow-up, and assumed that any subsequent result with the same RDT was also negative. It may be possible that, in some few instances, a subsequent RDT could have been positive. This definition may therefore have biased estimates of time to become negative towards shorter times.

## Conclusions

The ideal malaria RDT, a test that is both highly sensitive and highly specific in all epidemiological contexts is not yet available. A choice is to be made between an HPR2 test, with the risk of overdiagnosing malaria and thereby overlooking other possible causes of fever, and using a pLDH test, which carries the risk of missing true malaria cases with low parasitaemia. Three-band RDTs, combining detection of both HRP2 and pLDH antigens, are also available. However these RDTs are not better performing than the two-band tests [23] and may be a source of confusion when the HRP2 antigen is detected and not the pLDH one, or vice versa. In this sense an RDT detecting only pLDH with a higher sensitivity than the products currently on the market would be the optimal solution. Meanwhile, the findings of this study can be used to guide health decision makers in choosing the most appropriate test for a given context, and to help medical practitioners interpret RDT results.

## Additional files

10.1186/s12936-016-1529-6 Parasite densities by study site and sensitivity of three RDTs stratified by parasite density and study site.

10.1186/s12936-016-1529-6 Additional analysis of the time to become negative for the three malaria RDTs where patients experiencing recurrent parasitaemia were censored at the day when recurrent parasitaemia occurred.
