# Supplementary material for: Performance and time to become negative after treatment of three malaria rapid diagnostic tests in low and high malaria transmission settings
Source: Malar J. 2016 Oct 4;15:496. doi: 10.1186/s12936-016-1529-6 (PMC5050565; doi:10.1186/s12936-016-1529-6)
Supplement: Supplementary file 1 — 10.1186/s12936-016-1529-6 Parasite densities by study site and sensitivity of three RDTs stratified by parasite density and study site. [file 12936_2016_1529_MOESM1_ESM.pdf]

Additional file 1

article “**Performance and time to become negative after treatment of three malaria rapid diagnostic tests in low and high malaria transmission settings**”

Figure in Additional file 1 - Parasite densities by study site

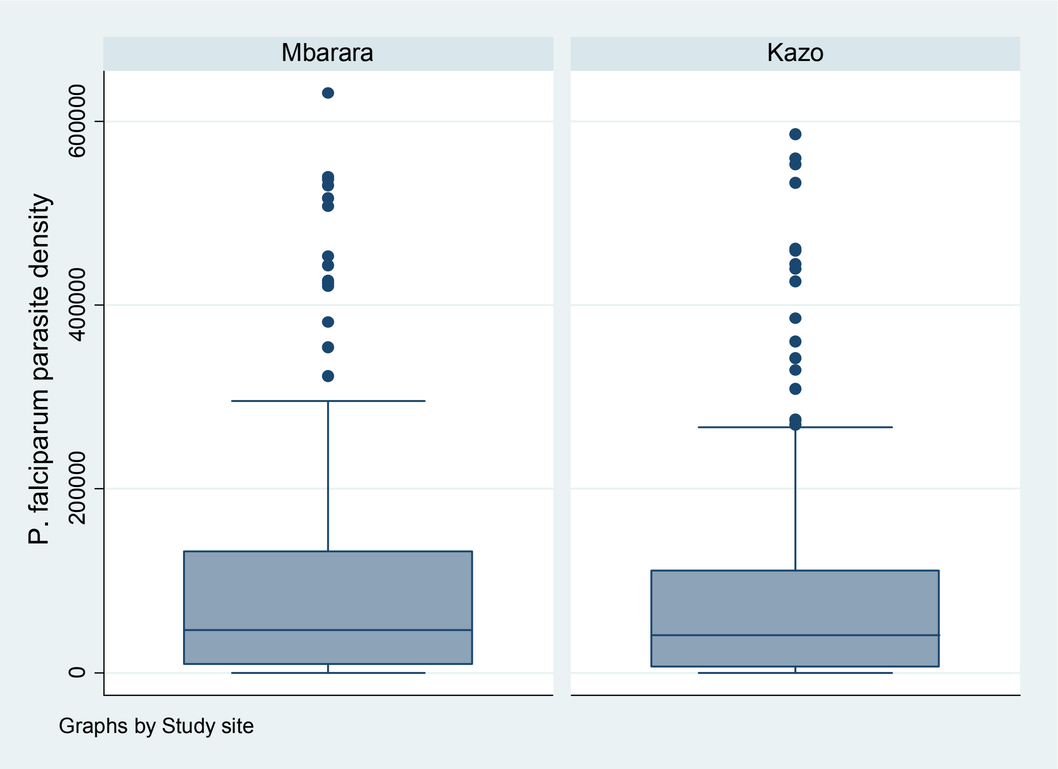

Table in Additional file 1 - Sensitivity of three RDTs stratified by parasite density and study site

Parasite density groups	Site	N	SD Bioline HRP2			CareStart HRP2			CareStart pLDH		
			n	%	[95% CI]	n	%	[95% CI]	N	%	[95% CI]
Low (1-1,999 parasites/µL)	Mbarara	31	28	90.3	[74.2-98.0]	29	93.5	[78.6-99.2]	23	74.2	[55.4-88.1]
	Kazo	42	41	97.6	[87.4-99.9]	40	95.2	[83.8-99.4]	32	76.7	[60.5-87.9]
Medium (2,000-199,999 parasites/µL)	Mbarara	200	200	100.0	[98.2-100.0]	200	100.0	[98.2-100.0]	199	99.5	[97.2-100]
	Kazo	186	185	99.5	[97.0-100.0]	185	99.5	[97.0-100.0]	182	97.8	[94.6-99.4]
High (200,000+ parasites/µL)	Mbarara	25	24	96.0	[79.6-99.9]	24	96.0	[79.6-99.9]	24	96.0	[79.6-99.9]
	Kazo	34	34	100.0	[89.7-100.0]	34	100.0	[89.7-100.0]	34	100.0	[89.7-100.0]
