# Supplementary material for: Performance and time to become negative after treatment of three malaria rapid diagnostic tests in low and high malaria transmission settings
Source: Malar J. 2016 Oct 4;15:496. doi: 10.1186/s12936-016-1529-6 (PMC5050565; doi:10.1186/s12936-016-1529-6)

Additional file 2

article "**Performance and time to become negative after treatment of three malaria rapid diagnostic tests in low and high malaria transmission settings**"

In this additional the same Kaplan-Meier survival analysis as presented in the main article is presented with the exception that patients with recurrent parasitaemia were included in the analysis and censored at the day when the recurrent parasitaemia occurred, instead of excluding them from the analysis.

This analysis included 197, 198 and 190 patients for SD Bioline HRP2, CareStart HRP2 and CareStart pLDH respectively in Mbarara and, in the same order, 197, 197 and 186 in Kazo.

The median time to become negative for SD Bioline HRP2 was 35 days in Mbarara and more than 42 days in Kazo. The median time to become negative for the CareStart HRP2 was more than 42 days in both sites. The median time to become negative for the CareStart pLDH was two days in both sites. The evolution of the time to become negative can be appreciated in the Figure below. Similar results were found in the analysis when patients with recurrent parasitaemia were excluded from the analysis.

Figure in Additional file 2 - Time to become negative for three malaria RDTs, expressed as Kaplan-Meier survival estimates, in Mbarara (low transmission setting) and in Kazo (high transmission setting). Patients with recurrent parasitaemia were censored at the day when recurrent parasitaemia occurred.

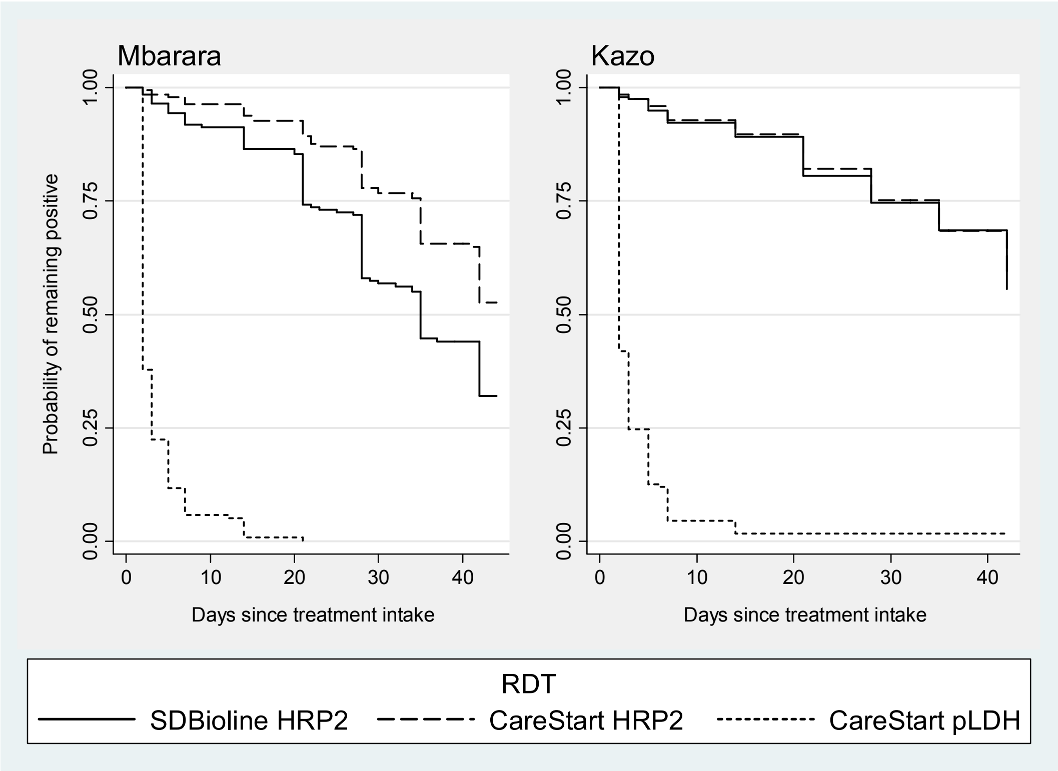

Supplement: Supplementary file 2 — 10.1186/s12936-016-1529-6 Additional analysis of the time to become negative for the three malaria RDTs where patients experiencing recurrent parasitaemia were censored at the day when recurrent parasitaemia occurred. [file 12936_2016_1529_MOESM2_ESM.pdf]
